# Blood Flow Restriction Training: To Adjust or Not Adjust the Cuff Pressure Over an Intervention Period?

**DOI:** 10.3389/fphys.2021.678407

**Published:** 2021-06-28

**Authors:** Mikhail Santos Cerqueira, Eduardo Caldas Costa, Ricardo Santos Oliveira, Rafael Pereira, Wouber Hérickson Brito Vieira

**Affiliations:** ^1^Neuromuscular Performance Analysis Laboratory, Department of Physical Therapy, Federal University of Rio Grande do Norte, Natal, Brazil; ^2^Department of Physical Education, Federal University of Rio Grande do Norte, Natal, Brazil; ^3^Integrative Physiology Research Center, Department of Biological Sciences, Universidade Estadual do Sudoeste da Bahia (UESB), Jequié, Brazil

**Keywords:** vascular occlusion exercise, kaatsu training, discomfort, perceived, pain, resistance training

## Abstract

Blood flow restriction (BFR) training combines exercise and partial reduction of muscular blood flow using a pressured cuff. BFR training has been used to increase strength and muscle mass in healthy and clinical populations. A major methodological concern of BFR training is blood flow restriction pressure (BFRP) delivered during an exercise bout. Although some studies increase BFRP throughout a training intervention, it is unclear whether BFRP adjustments are pivotal to maintain an adequate BFR during a training period. While neuromuscular adaptations induced by BFR are widely studied, cardiovascular changes throughout training intervention with BFR and their possible relationship with BFRP are less understood. This study aimed to discuss the need for BFRP adjustment based on cardiovascular outcomes and provide directions for future researches. We conducted a literature review and analyzed 29 studies investigating cardiovascular adaptations following BFR training. Participants in the studies were healthy, middle-aged adults, older adults and clinical patients. Cuff pressure, when adjusted, was increased during the training period. However, cardiovascular outcomes did not provide a plausible rationale for cuff pressure increase. In contrast, avoiding increments in cuff pressure may minimize discomfort, pain and risks associated with BFR interventions, particularly in clinical populations. Given that cardiovascular adaptations induced by BFR training are conflicting, it is challenging to indicate whether increases or decreases in BFRP are needed. Based on the available evidence, we suggest that future studies investigate if maintaining or decreasing cuff pressure makes BFR training safer and/or more comfortable with similar physiological adaptation.

## Introduction

Exercise with blood flow restriction (BFR) has been widely implemented in recent years (Patterson et al., 2019) due to increases in strength and muscle mass following low-load resistance training. Individuals with muscle weakness, such as patients with knee osteoarthritis (Cerqueira and de Brito Vieira, 2019), or who aim hypertrophy, such as athletes (Scott et al., 2016), can benefit from BFR training interventions. BFR is applied using a pneumatic cuff on the proximal aspect of the exercising limb. This cuff blocks venous return and partially occludes arterial blood flow, inducing increased metabolic stress (Suga et al., 2009).

Blood flow restriction pressure (BFRP) is a paramount aspect for exercise prescription with BFR. Historically, the first approach in BFRP application was to choose arbitrary absolute pressures (Takarada et al., 2000) or individualized cuff pressure at the lower limbs based on percentage of systolic blood pressure measured at the arms (Takano et al., 2005). Currently, it is known that BFRP needs to be individualized and adequate to limit muscle arterial blood flow partially with a recommended 40–80% of total restriction pressure (Patterson et al., 2019). Variables such as cuff width, limb circumference, ankle-brachial index, fat and muscle thickness, arterial stiffness, endothelial function, and blood pressure may influence BFRP (Loenneke et al., 2012, 2015; Hunt et al., 2016; Jessee et al., 2016; Bezerra de Morais et al., 2017). Doppler ultrasound (Bezerra de Morais et al., 2017), handheld Doppler (Laurentino et al., 2018), pulse oximetry (Zeng et al., 2019), and predictive equations (Hunt et al., 2016) have been proposed to adequately obtain and individually prescribe BFRP to maximize benefits and minimize discomfort/risks during BFR exercise (Singer et al., 2020; Spitz et al., 2020).

Nevertheless, no clear justification is provided in the literature for BFRP prescription during BFR exercises (Clarkson et al., 2020) and no specific recommendation of whether BFRP should be adjusted or not during a BFR training intervention. Previous studies reported daily or weekly adjustments increasing BFRP during 3–6 weeks of training in young males (Fahs et al., 2012; Crisafulli et al., 2018), while others did not adjust it in patients with musculoskeletal disorders (Bryk et al., 2016; Giles et al., 2017). Studies that observed increased BFRP (Fahs et al., 2012; Crisafulli et al., 2018) along intervention period probably considered relationships between limb circumference and BFRP, where greater gains in limb circumference following training required greater BFRP (Loenneke et al., 2015). However, increases in BFRP may not be imperative to induce neuromuscular adaptations (Lixandrão et al., 2015; Counts et al., 2016), whereas BFRP increments throughout training periods may not attenuate the perceived effort (Hughes et al., 2019).

Although neuromuscular adaptations are more studied, BFR training also promotes cardiovascular adaptations. For instance, arterial blood flow becomes turbulent during BFR exercise, altering central and peripheral hemodynamics (Takano et al., 2005; Hunt et al., 2016). After cuff deflation at the end of BFR exercise, ischemic reperfusion causes shear stress (Patterson and Ferguson, 2010; Hunt et al., 2013) and metabolic stimuli, which may affect vessel adaptations over time (Green et al., 2011). Also, BFR training can increase exercise pressor reflex, which is a reflex-mediated cardiovascular response, via engagement of the muscle metaboreflex (Sundblad et al., 2018; Cristina-Oliveira et al., 2020) and cardiac autonomic changes (Junior et al., 2019). Additionally, systolic blood pressure is directly proportional to BFRP (Loenneke et al., 2015; Bezerra de Morais et al., 2017), and studies showed SBP to reduce over weeks of BFR training (Cezar et al., 2016; Kambič et al., 2019).

Although important, the debate regarding magnitude of BFRP adjustments during training interventions is scarce (Clarkson et al., 2020). Considering that (1) the BFRP changes little over the course of training weeks (Mattocks et al., 2019), (2) the perceived effort tends to decrease throughout training with BFR (Martín-Hernández et al., 2017), and (3) the cardiovascular adaptations may reduce the required BFRP, increases in cuff pressure may unnecessarily cause discomfort, pain and risks (Loenneke et al., 2014; Spitz et al., 2020). In this sense, the present review aimed to discuss the need for BFRP adjustment based on cardiovascular outcomes and provide directions for future studies. Considering the well-established effects of BFR training on neuromuscular adaptations (Hughes et al., 2017; Centner et al., 2019), herein we focused on possible association between BFRP and cardiovascular adaptations induced by BFR training. Nevertheless, neuromuscular adaptations were addressed when necessary.

## Materials and Methods

An electronic search was conducted from inception to May 2021 on PubMed and Scopus databases to retrieve studies investigating the effects of BFR training on cardiovascular outcomes. Search strategy was restricted to English language literature, and the following terms were combined: “blood flow restriction,” “vascular occlusion,” “kaatsu training,” “exercise,” “training,” “chronic,” “vascular function,” “arterial stiffness,” “heart rate,” “metabolic stress,” and “blood pressure.” Forward and backward citations were conducted to locate further relevant titles. The following inclusion criteria were adopted: (a) assessment of chronic effects of BFR (>3 weeks training); (b) inclusion of outcomes that may influence directly or indirectly BFRP, such as macro- and microvascular function, arterial compliance, arterial stiffness and diameter, blood pressure, heart rate, and heart rate variability; (c) inclusion of humans older than 18-year-old. Study quality was assessed individually using PEDro scale (Maher et al., 2003).

## Cardiovascular Adaptations Induced by Blood Flow Restriction Training Interventions: What Do We Know So Far?

Structural adaptations in the vascular tree can occur due to metabolic changes elicited by hypoxia, mechanical stretch, shear stress, and increased growth factors, such as vascular endothelial growth factor (VEGF) (Hudlicka and Brown, 2009). BFR training can stimulate vascular adaptations, including severe hypoxia and increased expression of VEGF (Gustafsson et al., 1999; Takada et al., 2012). In addition, the reactive hyperemia occurring after BFR can stimulate capillarization (Evans et al., 2010). BFR training has been reported to improve arterial compliance and stiffness, as well as increase arterial diameter (Ozaki et al., 2011; Hunt et al., 2013). During BFR, there is an accumulation of metabolites that culminates in elevated metabolic stress as well as metaboreflex-induced increases in heart rate, blood pressure, and cardiac sympathetic modulation (Junior et al., 2019; Cristina-Oliveira et al., 2020). In the next sections, we discuss the main cardiovascular adaptations related to BFR training and how such adaptations could impact BFRP prescription during BFR training intervention. Table 1 summarizes the most common cardiovascular outcomes investigated during BFR training interventions and their potential impact on BFRP. A total of 29 studies met inclusion criteria and were included in this narrative review. Characteristics of the included studies and their main findings are shown in Table 2.

**TABLE 1:** Cardiovascular outcomes and their potential impacts on blood flow restriction pressure.

Outcomes	Definition	Measurement	Possible physiological repercussion	Possible repercussions on BFRP
FMD	Measurement of endothelial function. Defined as the artery dilatation in response to increases in blood flow	Doppler ultrasonography	Lower FMD indicates a reduction in endothelial function; increased FMD reflects an increase in reactive hyperemia	Lower FMD may be related to reduced vasodilatory response and increased BP and BFRP
Arterial compliance	The ability of an artery to expand and recoil during cardiac contraction and relaxation	PWV; CAVI; Doppler ultrasound	Its reduction is associated with increased BP; its increase is associated with reduced BP	Its reduction may result in increased BFRP; its increase may result in reduced BFRP
Arterial Stiffness	The decrease in arterial distensibility	PWV; CAVI	Its reduction may result in a reduced SBP; its increase may increase BP	Its reduction may result in a reduction of BFRP; its increase may result in increased BFRP
Arterial diameter	The straight line passing from side to side through the center of an artery	Doppler ultrasonography	Its reduction may result in increased SBP; its increase may result in a BP reduction.	Its reduction may result in increased BFRP; its increase may result in a reduced BFRP
Capillarity Index (microvascular filtration capacity)	An indirect measurement of the amount (expansion) of the capillary network	Plethysmography	Its reduction may result in increased peripheral vascular resistance and SBP; its increase may result in reduced peripheral vascular resistance and BP	Its reduction can result in increased BFRP; its increase may result in reduced BFRP
Blood pressure	The pressure of the blood against the inner walls of the blood vessels	Sphygmomanometer; Oscillometric device	It may be influenced by cardiac output, total peripheral resistance, and arterial stiffness.	Its reduction may result in reduced BFRP; its increase may result in increased BFRP
Heart rate	The number of times that the heart beats per minute	Electrocardiogram; Heart rate monitor	Its reduction may result in reduced BP; its increase may result in increased BP	Its reduction may result in reduced BFRP; its increase may result in increased BFRP
Heart rate variability	The variability of successive heart rate intervals	Heart rate monitor	Activation of baroreceptors leads to greater parasympathetic activity and sympathetic withdrawal	Increased parasympathetic activity and sympathetic withdrawal lead to hemodynamic adjusts, and increases in BFRP may be necessary

**FMD, flow-mediated dilation; PWV, pulse wave velocity; CAVI, cardio-ankle vascular index testing; BP, blood pressure; BRFP, blood flow restriction pressure.**

**TABLE 2:** Characteristics of the studies that investigated the effects of BFR training on cardiovascular outcomes.

Reference (year)	Sample (n)	Exercises protocol	Cardiovascular outcomes	BFR protocol	BFRP adjusted yes/no (reason)	Main findings
**Healthy young adults**						
Kim et al. (2009)	Young males (30)	Leg press, knee flexion, and knee extension (20% 1RM; 2 sets of 10 reps; 3x/week; 3 weeks)	AC, HR, SBP, and DBP	BFRP: 20% above systolic SBP; Cuff width: 5 cm	No (NR)	↔
Credeur et al. (2010)	Young males and females (20)	Handgrip (60% MVC; 15 grips/min; 20 min; 3x/week; 4 weeks)	FMD and AD	BFRP:80 mmHg; Cuff width: 4 cm	No (NR)	↓FMD and ↔ for other outcomes
Evans et al. (2010)	Young males (9)	Heel raises (30% MVC; 4 sets of 50 reps; 3x/week; 4 weeks)	MFC	BFRP:150 mmHg; Cuff width: 15 cm	No (NR)	↑MFC
Patterson and Ferguson (2010)	Young females (16)	Heel raises (25–50% 1RM; 3 sets until failure; 3x/week; 4 weeks)	RH	BFRP:110 mmHg; Cuff width: NR	No (NR)	↑RH
Clark et al. (2011)	Young males and females (16)	Knee extension (30% 1RM; 3 sets until failure; 3x/week; 4 weeks)	PWV	BFRP:30% above SBP; Cuff width: 6 cm	No (NR)	↔
Kacin and Strazar (2011)	Young males (10)	Knee extension (15% MVC; reps until failure; 4x/week; 4 weeks)	HR, SBP, and DBP	BFRP:230 mmHg; Cuff width: 13 cm	No (NR)	↑DBP and ↔ for other outcomes
Hunt et al. (2012)	Young males (9)	Handgrip (40% 1RM; 3 sets until failure; 3x/week; 4 weeks)	FMD and AD	BFRP:80 mmHg; Cuff width:13 cm	No (NR)	↑AD and ↔ for other outcomes
Fahs et al. (2012)	Young males (46)	Knee extension and knee flexion exercises (20% 1RM; 75 reps; 3x/week; 6 weeks)	AC, HR, SBP, DBP, and VCo	BFRP:160–200 mmHg; Cuff width: 5 cm	Yes (NR)	↑VCo ↓DBP and ↔ for other outcomes
Ozaki et al. (2013)	Young males (19)	Bench press (30% 1RM; 30 reps; 3x/week; 6 weeks)	AC, SBP, and DBP	BFRP:100–160 mmHg; Cuff width: 3 cm	Yes (to reduce arterial blood flow by 60% at rest and by 30% during exercise)	↔
Hunt et al. (2013)	Young males (11)	Heel raises (30% of 1RM; 3 sets until failure; 3x/week; 6 weeks)	FMD and AD	BFRP:110 mmHg; Cuff width: 13 cm	No (NR)	↑FMD ↑AD
Fahs et al. (2014)	Young males and females (16)	Knee extension (30% 1RM; 2–4 sets until failure;3x/week; 6 weeks)	PWV	BFRP: 80% of total restriction pressure; Cuff width: 5 cm	No (NR)	↑PWV
Crisafulli et al. (2018)	Young males (17)	Handgrip (30% MVC; 30 reps per minute for 3 min; 3x/week; 4 weeks)	HR and MAP	BFRP:75–150% of SBP; Cuff width: NR	Yes (NR)	↓MAP and ↔ for other outcomes
Mouser et al. (2019)	Young males and females (40)	Biceps curl and knee extension (15% 1RM; 4 sets of 90 reps; 2x/week; 8 weeks)	VCo	BFRP: 40 and 80% of total restriction pressure Cuff width: 5 cm	No (NR)	↑VCo
Christiansen et al. (2020)	Young males (10)	Interval cycling (61–81% of MAPO; 3 sets of three 2-min bouts; 3x/week; 6 weeks)	AD	BFRP: 180 mmHg; Cuff width: 13 cm	No (NR)	↑AD
Early et al. (2020)	Young males and females (31)	Biceps curl, elbow extension, knee extension, leg curl and heel raise (30% 1RM; 3sets of 30 reps; 2–3x/week; 8 weeks	HR, SBP, DBP, and FMD	BFRP: 250 or 350 mmHg Cuff width: 5.5 or 7.0 cm	No (NR)	↓SBP ↑FMD and ↔ for other outcomes
Ramis et al. (2020)	Young males (28)	Elbow flexion and knee extension (30% 1RM; 4 sets of 21–23 reps; 3x/week; 8 weeks)	FMD	BFRP: 20 mmHg below SBP; Cuff width: 14–16 cm	No (NR)	↑FMD
Zhao et al. (2020)	Young males (24)	Elbow flexion (30% 1RM; 5 sets of 20 reps; 5xweek; 8 weeks)	HR, SBP, DBP, and MAP	BFRP: 65 or 130% of SBP; Cuff width: NR	No (NR)	↓HR and ↔ for other outcomes
**Healthy middle-aged and older adults**						
Ozaki et al. (2011)	Older males and females (23)	Treadmill walking (45% of heart rate reserve; 20 min; 4x/week; 10 weeks)	AC, HR, SBP, and DBP	BFRP:140–200 mmHg; Cuff width: 5 cm	Yes (to impose progressive overload in muscle and central circulation)	↑AC and ↔ for other outcomes
Patterson and Ferguson (2011)	Older males and females (10)	Heel raises (25% 1RM; 3 sets until failure;3x/week; 4 weeks)	RH	BFRP:110 mmHg; Cuff width: NR	No (NR)	↑RH
Yasuda et al. (2014)	Older males and females (19)	Knee extension and leg press (20–30% 1RM; 75 reps; 2x/week; 12 weeks)	FMD, HR, SBP, DBP, and AS	BFRP:120–270 mmHg; Cuff width: 5 cm	Yes (to achieve high levels of perceived effort).	↑FMD and ↔ for other outcomes
Yasuda et al. (2015a)	Older females (14)	Arm curl and triceps down (elastic resistance; 75 reps; 2x/week; 12 weeks)	HR, SBP, DBP, FMD, and AS	BFRP:120–270 mmHg; Cuff width: 3 cm	Yes (to achieve high levels of perceived effort).	↔
Yasuda et al. (2015b)	Older males and females (17)	Arm curl and triceps down (Elastic resistance; 75 reps; 2x/week; 12 weeks)	HR, SBP, DBP, FMD, and AS	BFRP:120–270 mmHg; Cuff width: 3 cm	Yes (to achieve high levels of perceived effort).	↔
Shimizu et al. (2016)	Older males and females (40)	Leg extension, leg press, rowing and chest press (20% 1RM; 3 × 20 reps; 3x/week; 4 weeks)	RH	BFRP: 100% of SBP Cuff width: 10 or 7 cm	No (NR)	↑RH
Kim et al. (2017)	Young and older males and females (27)	Handgrip (20% MVC; 3 sets until failure; 2x/week; 4 weeks)	VCo, HR, SBP, and DBP	BFRP:130% of SBP; Cuff width: 16 cm	No (NR)	↑VCo and ↔ for other outcomes
Junior et al. (2019)	Middle-aged males (21)	Treadmill walking (6 km⋅h^–1^, 5% grade; 5 sets of 3 min; 3x/week; 6 weeks)	HR and HRV	BFRP:80–100 mmHg; Cuff width: 18 cm	Yes (to allow participants to adapt to the BFRP during the early phase of the training)	↓HR ↑HRV
Ferreira Junior et al. (2019)	Middle-aged males (21)	Treadmill walking (6 km⋅h^–1^, 5% grade; 5 sets of 3 min; 3x/week; 6 weeks)	SBP, DBP, and HRV	BFRP:80–100 mmHg; Cuff width: 18 cm	Yes (to allow participants to adapt to the BFRP during the early phase of the training)	↑HRV ↓SBP and ↔ for other outcomes
**Clinical populations**						
Cezar et al. (2016)	Hypertensive females (23)	Wrist flexion (30% MVC; 3 sets until failure; 2x/week; 8 weeks)	HR, SBP, and DBP	BFRP:70% of SBP; Cuff width: NR	Yes; based on SBP (to adjust BFRP to SBP in each training session).	↓SBP ↓DBP and ↔ for other outcomes
Barbosa et al., 2018	Patients with chronic kidney disease	Handgrip (40% MVC; 3 sets of 20 reps; 2x/week; 8 weeks)	AD	BFRP:50% of SBP; Cuff width: NR	No (NR)	↑AD
Kambič et al. (2019)	Patients with coronary artery disease (24)	Knee extensions (30–40% 1RM; 3 sets of 8–12 reps; 2x/week; 8 weeks)	HR, SBP, and DBP	BFRP:15–20 mmHg above systolic BP; Cuff width: 23 cm	Yes (NR)	↓SBP and ↔ for other outcomes

**AC, arterial compliance; HR, heart rate; SBP, systolic blood pressure; DBP, diastolic blood pressure; BFRP, blood flow restriction pressure; MVC, maximal voluntary contraction; MAPO, maximal aerobic power output; FMD, flow-mediated dilation; AD, arterial diameter; MFC, microvascular filtration capacity; RH, reactive hyperemia; PWV, pulse wave velocity; VCo, vascular conductance; MAP, mean arterial pressure; AS, arterial stiffness; HRV, heart rate variability; NR, not reported; ↔ no significant differences; ↑ increased; ↓ decreased.**

### Macro- and Microvascular Function

Macro- and microvascular function were investigated during BFR training interventions using measures of flow-mediated dilation (FMD), peak of post-occlusive blood flow (reactive hyperemia), and capillarity index. FMD is a measure of NO-dependent endothelial function (i.e., artery dilation following increased blood flow) (Kelm, 2002), calculated as the difference in arterial diameter between pre- and post-blood flow occlusion. Reactive hyperemia and capillarity index are usually measured using plethysmography. The former is defined as the transient increase in blood flow following a period of ischemia (arterial occlusion) (Patterson and Ferguson, 2010) and may be related to capillarity index, an indirect marker of microvascular network expansion that can facilitate muscle oxygen delivery (Evans et al., 2010; Hunt et al., 2013).

The effect of BFR training on macro- and microvascular function is contradictory. Hunt et al. (2013) observed increased popliteal artery FMD of healthy young males 2–4 weeks following unilateral plantar flexion training with BFR (30% of one-repetition maximum [1RM]; 3 sets until failure; 3x/week; BFRP 110 mmHg). Similarly, increased brachial artery FMD was found in healthy young adults following 8 weeks of upper and lower limbs exercises (30% 1RM; 3–4 sets of 21–30 reps; 3x/week; BFRP between 120 and 270 mmHg or based on SBP) (Early et al., 2020; Ramis et al., 2020); and healthy older adults following 12 weeks of knee extension and leg press exercises with BFR (20–30% 1RM; 75 reps; 2x/week; BFRP between 120 and 270 mmHg) (Yasuda et al., 2014). In contrast, no significant changes were observed in brachial artery FMD following handgrip training with BFR (40% 1RM until failure; 3x/week for 3 weeks; BFRP 80 mm Hg) in healthy young individuals (Hunt et al., 2012) or arm training with BFR in older adults (elastic resistance; 75 reps; 2x/week; BFRP between 120 and 270 mmHg) (Yasuda et al., 2015a,b).

Interestingly, Credeur et al. (2010) showed a significant reduction in brachial artery FMD in healthy young adults following handgrip training with BFR [60% of maximal voluntary contraction (MVC); 15 grips per minute during 20 min; 3x/week for 4 weeks; BFRP 80 mmHg]. This finding suggests reduced vasodilatory capacity, a marker of vascular function. These results may be partially explained by both the high load (60% of MVC) and prolonged BFR (20 min) used, which were greater than other protocols (20–30% 1RM, 5–10 min) (Hunt et al., 2013; Yasuda et al., 2014). It can be argued that the combination of high load and prolonged BFR may increase oxidative stress (Biazon et al., 2019) and lead to endothelium dysfunction through decreased nitric oxide bioavailability (Förstermann, 2006). Age differences between participants and both the duration and training intensity may also explain contradictory findings among studies.

Regarding reactive hyperemia, increased post-occlusive calf blood flow following low load plantar flexion training with BFR for 4–6 weeks (25–50% 1RM until failure; 3x/week; BFRP 110 mm Hg) was found in young and older adults (Patterson and Ferguson, 2010, 2011; Hunt et al., 2013). Shimizu et al. (2016) demonstrated increased reactive hyperemia in older adults following multi-exercise (leg extension, leg press, rowing, and chest press) BFR training for 4 weeks (20% 1RM; 3 sets of 20 reps; 3x/week; BFRP equivalent to systolic blood pressure). Similarly, Fahs et al. (2012) observed an increase in the calf blood flow of healthy young males following 6 weeks of knee extension and flexion BFR training (20% 1RM; 75 reps; 3x/week; BFRP 160–200 mmHg). Mouser et al. (2019) also found increased forearm and calf vascular conductance in healthy young adults following 8 weeks of biceps curl and knee extension exercises (15% 1RM; 4 sets of 90 reps; 2x week; BFRP 40 or 80% of total restriction pressure). In healthy young adults Kim et al. (2017) showed increased forearm vascular conductance (i.e., blood flow/mean arterial pressure) following 4 weeks of isometric handgrip training with BFR (20% MVC until failure; 3x/week; BFRP 130% of systolic blood pressure). The above-mentioned improvements on vascular function following BFR training may also reflect improvements in microvascular tree, such as increased muscle capillarity (Hodges et al., 2010; Patterson and Ferguson, 2010). Calf capillary filtration capacity, an indirect capillarity index, also increased 14–26% following 4–6 weeks of unilateral calf raise or plantar flexion training with BFR (30% MVC; 4 sets of 50 reps or to failure; 3x/week; BFRP 110–150 mmHg) in young adult males (Evans et al., 2010; Hunt et al., 2013).

Overall, the possible increase in vasodilatory capacity to reactive hyperemia after BFR training may impact BFRP applied during exercise. For example, increased vasodilatory capacity may indicate a reduction in peripheral vascular resistance (Satoh, 2011; Hunt et al., 2013); however, contradictory findings preclude the recommendation of a reduction in BFRP during the training period based on macro- and microvascular adaptations.

### Arterial Compliance, Stiffness, and Diameter

In studies involving BFR training, arterial stiffness and/or compliance are assessed non-invasively using pulse wave velocity (PWV), cardio-ankle vascular index testing (CAVI), or Doppler ultrasonography (Hunt et al., 2013; Yasuda et al., 2014; Karabulut et al., 2018). PWV is defined as the velocity at which pressure wave propagates along the arterial tree (Pereira et al., 2015), and lower PWV values indicate reduced arterial stiffness. CAVI is obtained by recording the distance from the level of the aortic valve to the ankle and the time delay between closure of aortic valve and detected change in arterial pressure wave at the ankle level (Yambe et al., 2004). Higher CAVI values indicate greater arterial stiffness (Sun, 2013).

Studies addressing arterial compliance and stiffness adaptations following BFR training are also controversial. Clark et al. (2011) did not observe changes in lower limb PWV of healthy young males following 4 weeks of bilateral knee extension BFR training (30% 1RM; 3 sets until failure; 3x/week; BFRP 1.3x above systolic blood pressure). No significant changes were observed in brachial artery stiffness of healthy older adults following 12 weeks of either knee extension and leg press BFR training (20–30% 1RM; 2x/week; BFRP between 120 and 270 mmHg) (Yasuda et al., 2014) or arm curl and triceps down BFR training (elastic resistance; 2x/week; BFRP 120 and 270 mmHg) (Yasuda et al., 2015b). Furthermore, no significant changes were found in brachial artery compliance of healthy young males following 3–6 weeks of upper and lower limb BFR training (20–30% 1RM; 20–75 reps; 3x/week; BFRP between 100 and 200 mmHg) (Kim et al., 2009; Fahs et al., 2012; Ozaki et al., 2013).

Fahs et al. (2014) observed an increase in peripheral PWV of middle-aged individuals following 6 weeks of knee extension BFR training (30% 1RM; 2–4 sets until failure; 3x/week; BFRP 50–80% of total restriction pressure). Specific effects of BFR on arterial compliance and stiffness, when different types of exercise training (aerobic vs. resistance) are performed, are still unknown. However, adaptations in these outcomes following aerobic or resistance training with BFR seem to be comparable to training without BFR. Therefore, based on these conflicting results, it is unclear whether arterial stiffness and compliance may impact the need for adjustments in BFRP.

Few data are available regarding the effect of BFR training on arterial diameter. Enlargement of brachial and radial artery was reported following 4–8 weeks of handgrip BFR training in young adult males (40% 1RM; 3 sets until failure; 3x/week; BFRP 80 mmHg) (Hunt et al., 2012) and patients with chronic kidney disease (40% MVC; 3 sets of 8–12 reps; 2x/week; BFRP 50% of SBP) (Barbosa et al., 2018). Similarly, Hunt et al. (2013) found an increase in popliteal artery diameter following 6 weeks of plantar flexion BFR training (30% 1RM until failure, 3x/week; BFRP 110 mmHg) in healthy males. Also, increased femoral artery diameter was found in young males following 6 weeks of interval cycling training (61–81% of maximal aerobic power output; 3 sets of three 2-min bouts; 3x/week) (Christiansen et al., 2020). Conversely, Credeur et al. (2010) observed no significant changes in brachial artery diameter of healthy young adults following 4 weeks of handgrip BFR training (60% of MVC; 15 grips per minute during 20 min; 3x/week; BFRP 80 mmHg). In summary, resistance BFR training may increase arterial diameter of upper and lower limbs, except following long sets of training (20 min). More data are needed to better understand the impact of aerobic BFR training interventions on arterial diameter.

### Heart Rate and Blood Pressure

While heart rate (HR) and blood pressure (BP) increase during BFR exercise (Takano et al., 2005), adaptations in resting HR and BP following BFR training intervention are contradictory. For instance, Ozaki et al. (2011, 2013) did not observe changes in HR and BP following either 10 weeks of BFR walking training (20 min at 45% of heart rate reserve; 4x/week) or 6 weeks of bench press BFR training (30% 1RM; 3x/week; BFRP between 100 and 200 mmHg). Similarly, no significant changes in HR and BP were found in healthy older adults following BFR resistance training involving lower and upper limbs (20–30% 1RM or elastic resistance; 75 reps; 2x/week; BFRP between 120 and 270 mmHg) (Yasuda et al., 2014, 2015b). In young adults, no significant changes were observed in HR and BP following 3 weeks of leg press, knee flexion and knee extension training with BFR (20% 1RM; 2 sets of 10 reps; 3x/weeks; BFRP 20% above systolic BP) (Kim et al., 2009).

In contrast, Junior et al. showed a reduction in HR of healthy males following 6 weeks of BFR walking training (6 km⋅h^–1^, 5% grade; 5 sets of 3 min; 3x/week; BFRP between 80 and 100 mmHg) (Junior et al., 2019). Similarly, reduced mean BP was observed in healthy young individuals following 4 weeks of BFR handgrip training (30 contractions per minute at 30% MVC; 3x/week; BFRP between 75 and 150% of systolic blood pressure) (Crisafulli et al., 2018) and 8 weeks of BFR elbow flexion training (30% 1RM; 5 sets of 20 reps; 5x/week; BFRP 65 of 130% of SBP) (Zhao et al., 2020). Fahs et al. (2012) also demonstrated a reduced diastolic BP of healthy young males following 6 weeks of BFR training with knee extension and flexion (75 reps with 20% 1RM; 3x/week; BFRP between 160 and 200 mmHg). An unexpected increase in diastolic BP was observed following 4 weeks of knee extension BFR training (15% MVC; reps until failure; 4x/week; BFRP 230 mmHg) in healthy young individuals (Kacin and Strazar, 2011).

Based on preliminary findings, BFR training has a BP-lowering effect in clinical populations. For example, Cezar et al. (2016) showed a reduction in systolic BP, diastolic BP, mean BP and double product of hypertensive women following 8 weeks of wrist flexion BFR training (30% of MVC; 3 sets until failure; 2x/week; BFRP 70% of systolic BP). More recently, Kambič et al. (2019) observed a reduced systolic BP of patients with coronary artery disease following 8 weeks of knee extension BFR training (30–40% 1RM; 3 sets of 8–12 reps; 2x/week; BFRP ∼140 mmHg). Lastly, two studies verified an increase in HR variability (Ferreira Junior et al., 2019; Junior et al., 2019). Although literature is inconsistent, there is evidence to support that BFR training may reduce both HR and BP and increase HR variability in clinical populations.

## Is Cuff Pressure Adjusted in Blood Flow Restriction Training Interventions?

Most included studies were performed with young adults, middle-aged and older adults, with or without clinical conditions, and used lower limb exercises (i.e., leg press, knee extension and flexion, or heel raises) (Kim et al., 2009; Evans et al., 2010; Patterson and Ferguson, 2010, 2011; Clark et al., 2011; Kacin and Strazar, 2011; Fahs et al., 2012, 2014; Hunt et al., 2013; Yasuda et al., 2014; Kambič et al., 2019). Studies also used upper limb exercises (i.e., handgrip, elbow flexion, bench press, or triceps down) (Credeur et al., 2010; Hunt et al., 2012; Ozaki et al., 2013; Yasuda et al., 2015b; Cezar et al., 2016; Kim et al., 2017; Crisafulli et al., 2018) and treadmill walking (Ferreira Junior et al., 2019; Junior et al., 2019). Importantly, many of the included studies had methodological limitations, such as failure in allocation concealment, blinding of participants and assessors; and intention-to-treat analysis (Table 3).

**TABLE 3:** Methodological quality of eligible studies (*n* = 29).

Study	PEDro scale items^a^											PEDro score
	
	1^b^	2	3	4	5	6	7	8	9	10	11	(0–10)
Kim et al. (2009)	Y	Y	N	Y	N	N	Y	Y	Y	Y	Y	7
Credeur et al. (2010)	Y	Y	N	Y	N	N	N	Y	Y	Y	Y	6
Evans et al. (2010)	Y	N	N	Y	N	N	N	Y	Y	Y	Y	5
Patterson and Ferguson (2011)	N	N	N	Y	N	N	N	Y	Y	Y	Y	5
Clark et al. (2011)	N	Y	N	Y	N	N	Y	Y	Y	Y	Y	7
Kacin and Strazar (2011)	N	N	N	Y	N	N	N	Y	Y	Y	Y	5
Ozaki et al. (2011)	N	N	N	Y	N	N	Y	Y	Y	Y	Y	6
Patterson and Ferguson (2011)	N	N	N	Y	N	N	N	Y	Y	Y	Y	5
Hunt et al. (2012)	N	N	N	Y	N	N	N	Y	Y	Y	Y	5
Fahs et al. (2012)	Y	Y	N	Y	N	N	N	Y	Y	Y	Y	6
Ozaki et al. (2013)	N	Y	N	Y	N	N	Y	Y	Y	Y	Y	6
Hunt et al. (2013)	N	Y	N	Y	N	N	Y	Y	Y	Y	Y	7
Fahs et al. (2014)	N	Y	N	Y	N	N	N	Y	N	Y	Y	5
Yasuda et al. (2014)	Y	Y	N	Y	N	N	N	N	Y	Y	Y	6
Yasuda et al. (2015a)	N	N	N	Y	N	N	N	Y	Y	Y	Y	5
Yasuda et al. (2015b)	N	Y	N	Y	N	N	N	Y	Y	Y	Y	6
Cezar et al. (2016)	Y	Y	Y	Y	N	N	N	Y	N	Y	Y	6
Shimizu et al. (2016)	Y	Y	N	Y	N	N	N	Y	Y	Y	Y	6
Kim et al. (2017)	Y	Y	N	Y	N	N	N	N	N	Y	Y	4
Barbosa et al., 2018	Y	Y	Y	Y	N	N	N	N	Y	Y	Y	6
Crisafulli et al. (2018)	N	Y	N	Y	N	N	N	N	N	Y	Y	4
Ferreira Junior et al. (2019)	N	Y	N	Y	N	N	N	Y	N	Y	Y	5
Junior et al. (2019)	N	Y	N	Y	N	N	N	Y	N	Y	Y	5
Kambič et al. (2019)	Y	Y	Y	N	N	N	N	Y	Y	Y	Y	6
Mouser et al. (2019)	N	Y	N	Y	N	N	N	Y	N	Y	Y	5
Christiansen et al. (2020)	N	Y	N	Y	N	N	Y	N	N	Y	Y	5
Early et al. (2020)	N	Y	N	Y	N	N	N	Y	N	Y	Y	5
Ramis et al. (2020)	Y	Y	N	Y	N	N	Y	Y	N	Y	Y	6
Zhao et al. (2020)	N	Y	N	Y	N	N	N	Y	Y	Y	Y	6

*Y, Yes; N, no.*

*^*a*^1: Eligibility criteria and source of participants; 2: random allocation; 3: concealed allocation; 4: baseline comparability; 5: blinded participants; 6: blinded therapists; 7: blinded assessors; 8: adequate follow-up; 9: intention-to-treat analysis; 10: between-group comparison; 11: point estimates and variability.*

*^*b*^Item 1 does not contribute to total score.*

Overall, 35% (*n* = 10) of the included studies adjusted BFRP during BFR training intervention; of these, three did not provide clear justification (Fahs et al., 2012; Crisafulli et al., 2018; Kambič et al., 2019). Rationale and methods for BFRP adjustments during BFR training interventions are diverse. Those who justified, argued that adjustments were performed to achieve high levels of perceived effort (Yasuda et al., 2014, 2015b), allow participants to adapt to the occlusion stimulus during the early phase of training (Ferreira Junior et al., 2019; Junior et al., 2019), impose progressive overload in skeletal muscles and central circulation (Ozaki et al., 2011), adjust BFRP to systolic BP in each session (Cezar et al., 2016), or reduce arterial blood flow by 60% at rest and 30% during exercise (Ozaki et al., 2013).

In three studies, BFRP adjustment was based on systolic BP changes (75–150% of the systolic BP) (Cezar et al., 2016; Crisafulli et al., 2018; Kambič et al., 2019), whereas six studies adjusted it arbitrarily by applying absolute restriction pressures ranging from 80 to 270 mmHg (Ozaki et al., 2011, 2013; Fahs et al., 2012; Yasuda et al., 2015b; Ferreira Junior et al., 2019; Junior et al., 2019). Applying arbitrary or SBP-based cuff pressures has not encouraged (Patterson et al., 2019). In fact, an absolute pressure of 160 mmHg in the upper limbs would result in partial arterial occlusion in some participants and in total arterial occlusion in others (Loenneke et al., 2013). As an alternative, researchers can use BFRP relative to PAS. However, the resulting pressure of this strategy may vary as a percentage of BFRP due to different cuff width/type (Loenneke et al., 2012).

Variability related to cuff width was also present in BFR protocols. For example, cuff width ranged from 3 to 16 cm for upper limb exercises (Ozaki et al., 2013; Kim et al., 2017) and from 5 to 23 cm for lower limbs (Yasuda et al., 2014; Kambič et al., 2019). Four of the included studies did not report cuff width. Although wide BFR cuffs restrict arterial blood flow at a lower pressure than narrow BFR cuffs (Loenneke et al., 2012), wider cuffs are associated with higher rate of perceived effort than narrow ones when the same arbitrary arterial occlusion pressure is prescribed for all participants (Rossow et al., 2012). Considering that high rates of perceived exertion can impair adherence to exercise (Chao et al., 2000), the influence of cuff width on rate of perceived exertion and discomfort should be investigated in future studies regarding BFRP adjustments (Spitz et al., 2020).

## Is There a Need to Adjust Blood Flow Restriction Pressure During a Blood Flow Restriction Training Intervention?

A common limitation of BFR training interventions is the variety of methods to prescribe BFRP. A broad range of cuff pressures has been used (Patterson et al., 2019; Clarkson et al., 2020), with few studies providing rationale for BFRP adjustment during BFR training interventions. Also, time under BFR is a variable that can influence cardiovascular adaptations and is inconsistent across studies. In fact, vascular function reduced when the time under BFR was 20 min (Credeur et al., 2010), while shorter times under BFR (∼11 min) improved vascular function (Yasuda et al., 2014). Training protocol also varied among studies and involved a wide range of training weeks, resistance exercise (dynamic or isometric) or aerobic exercise for the upper limbs, lower limbs, or both. This is relevant because cardiovascular adaptations to exercise can be influenced by modality (aerobic vs. resistance), frequency, intensity, time or duration, and volume (Green et al., 2011; Vanhees et al., 2012).

Of the 15 studies that assessed BP and/or HR following BFR training interventions, 12 used low-load resistance exercises with BFR, whereas three studies used treadmill walking protocols. Overall, no significant changes in HR were found in young participants, while diastolic BP was significantly reduced (−7 mmHg) in one study (Fahs et al., 2012) and increased (7 mmHg) in another study (Kacin and Strazar, 2011). In middle-aged and older adults, one study found a significant decrease in systolic BP (−10 mmHg) (Ferreira Junior et al., 2019), and another observed a decrease in HR (−10 bpm) (Junior et al., 2019). In clinical populations, two studies observed decreased systolic BP (−6 to −16 mm Hg) (Cezar et al., 2016; Kambič et al., 2019), and one study found a decreased diastolic BP (−11 mmHg) (Cezar et al., 2016). Thus, based on preliminary findings, it seems that BFR training may elicit BP-lowering effect and arterial diameter enlargement (Hunt et al., 2012, 2013; Barbosa et al., 2018; Christiansen et al., 2020).

Considering the isolated influence of cardiovascular factors (especially systolic BP) on BFRP (Loenneke et al., 2015; Bezerra de Morais et al., 2017), it seems plausible that a reduction in BFRP during BFR training period may be applied to minimize the discomfort caused by compression of the active musculature without compromising efficacy of the training method. However, in a more holistic perspective, the possible BFRP-lowering induced by cardiovascular adaptations may just compensate possible BFRP-increasing induced by neuromuscular adaptations (i.e., increased limb circumference secondary to muscle hypertrophy). Therefore, from a broader perspective, maintaining a percentage of BFRP levels seems the most plausible approach. In fact, the percentage of BFRP does not change appreciably over the course of a training intervention in healthy young (Mattocks et al., 2019), however this issue has not been investigated in clinical populations.

It is important to note that there is a minimum and maximum BFRP threshold (40–80% of total restriction pressure) recommended to induce neuromuscular adaptations (Patterson et al., 2019). In this sense, especially when working at the lower threshold (40% of total restriction pressure), periodic monitoring of BFRP may be important to reduce “low doses” of BFR. In addition, 40–80% of total restriction pressure is suggested to stimulate neuromuscular adaptations; however, minimum and maximum BFRP threshold to induce cardiovascular adaptations is not yet established. Based on a limited number of studies, a recent meta-analysis found no differences between high or low BFRP on vascular function compared to exercise without BFR (Liu et al., 2021). Nevertheless, the impact of different BFR pressures on adaptations in other cardiovascular variables, such as BP, heart rate and arterial diameter is unclear.

Of the 10 studies examining cardiovascular outcomes, only seven justified BFRP adjustments. Importantly, some studies (Yasuda et al., 2014, 2015b) reached extremely high pressures(e.g., 270 mmHg). If the applied BFRP increases as training progress, pain perception also increases (Weatherholt et al., 2013). Interestingly, to induce neuromuscular adaptations higher relative BFRP may not be necessary when exercising under BFR with loads of 30–40% 1RM (Lixandrão et al., 2015; Counts et al., 2016), whereas higher pressures (80% of total restraint pressure) may be required when exercising with lower loads (20% 1RM) (Lixandrão et al., 2015). This debate is relevant because perceived effort and pain may decrease without impairing neuromuscular gains over training weeks when fixed BFR is applied (Martín-Hernández et al., 2017; Teixeira et al., 2020), but perceived effort is not attenuated if pressure increases (Hughes et al., 2019). Thus, unnecessary increases in BFRP may contribute to discomfort, impair adherence (Spitz et al., 2020), and increase the risk of adverse events (Bond et al., 2019), such as venous thromboembolism, rhabdomyolysis, and bruising (Patterson et al., 2019; Whiteley, 2019).

## Conclusion and Future Perspectives

In summary, cardiovascular adaptations induced by BFR training are conflicting. Based on the current literature, it is difficult to indicate whether increase or decrease BFR is needed, particularly in short-term BFR training interventions (i.e., < 6 weeks). Studies investigating whether BFRP adjustments throughout training are more effective, safe and/or tolerable than maintaining constant pressures are needed. Moreover, individualized and validated measures of BFRP, such as ultrasound Doppler (Bezerra de Morais et al., 2017), handheld Doppler (Laurentino et al., 2018), or pulse oximetry (Zeng et al., 2019) are encouraged. We also suggest that future studies investigate whether there is a minimum and maximum BFRP threshold to induce cardiovascular adaptations. Finally, considering possible BP-lowering effect of BFR training, longitudinal studies directly measuring BFRP are needed to establish whether cuff pressure is altered in clinical populations.

## Author Contributions

MSC conceived the study. MSC, EC, RS, RP, and WB participated to the design and coordination and wrote the manuscript. All authors have read and approved the final version of the manuscript, and agreed with the order of presentation of the authors.

## Conflict of Interest

The authors declare that the research was conducted in the absence of any commercial or financial relationships that could be construed as a potential conflict of interest.
